# Radiation Pneumonitis after Radiotherapy of Neck Lymphoma

**DOI:** 10.1155/2014/614984

**Published:** 2014-08-28

**Authors:** Min Wei, Jun Cai, Tao Tong, Shihua Yu, Yonghua Yang, Weijia Zhang, Jiyuan Yang

**Affiliations:** Department of Oncology, First Affiliated Hospital of Yangtz University, Hubei, Jingzhou 434000, China

## Abstract

Radiotherapy is still one of the effective means for treatment of malignant tumors up to now. Particularly, it is an indispensable effective measure for treatment of some lymphoma patients. In routine work, radiation pneumonitis (RP) is the most significant complication of acute treatment-related toxicities in lung cancer; however, serious radioactive pneumonia is rare for the radiotherapy of neck lymphoma because the volume of the lungs affected by radiation dose was very small. We report a lymphoma case, where the patient had undergone radiotherapy for the bilateral neck and bilateral supraclavicular/infraclavicular area. Following completion of radiotherapy, the patient developed severe radiation pneumonitis.

## 1. Clinical Data

**Figure 1:**
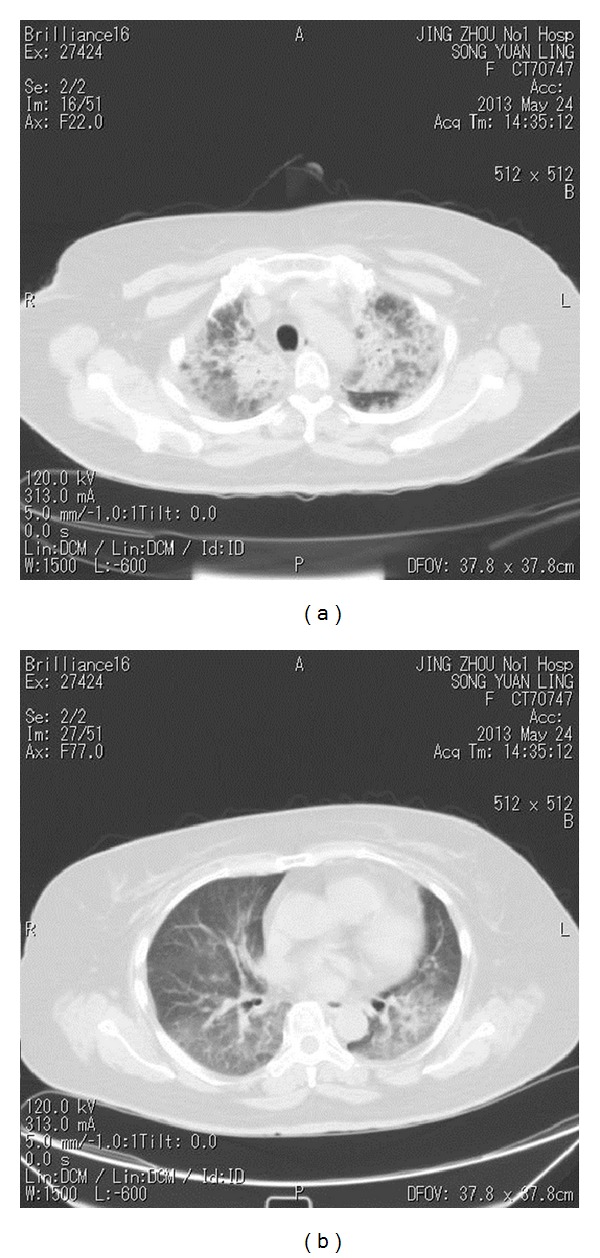


**Figure 2:**
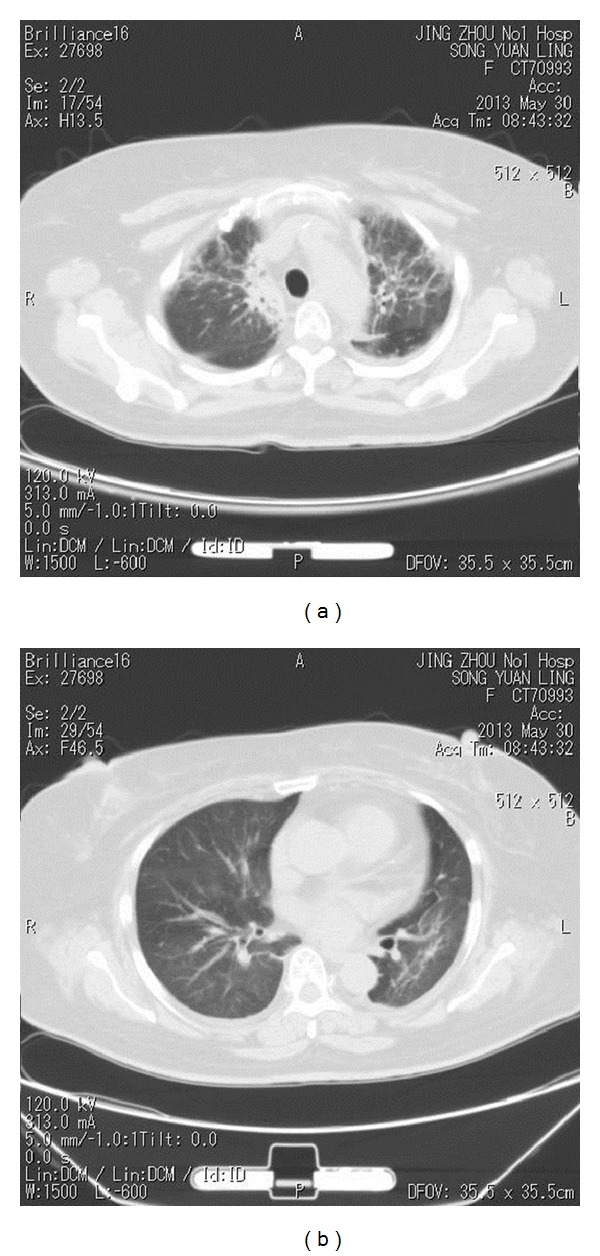


A 54-year-old female patient presented with a progressive enlarging mass in the right neck for more than two months, associated with difficulties in breathing and swallowing for one week. The patient does not smoke and has no diabetes history. A neck magnetic resonance imaging (MRI) showed space-occupying lesion in the right lobe of the thyroid, combined with bilateral cervical and supraclavicular/infraclavicular multiple enlarged lymph nodes, and the diameter of the biggest one was 6 cm. The biopsy of one of the enlarged lymph nodes showed diffuse large B-cell lymphoma of non-Hodgkin's lymphoma, and the immunohistochemical results were as follows: CD3 (−), CD10 (−), CD20 (+), CD30 (−), Mum-1 (+), CyclinD (−), ALK (−), bcl-2 (+), CD21 weak (+), and Ki67 (+) > 70~80%. The whole-body positron emission tomography-computed tomography scan (PET-CT) showed “right thyroid, bilateral cervical, and bilateral supraclavicular/infraclavicular nodules with increased metabolism, and the range of standard uptake values (SUVs) is between 3.5 and 4.5, suggesting a great possibility of malignancy.” The bone marrow aspiration cytology showed no malignant cells. A clinical diagnosis of non-Hodgkin's lymphoma was made and the clinical stage was at IIA. The patient had been given the CHOP regiment (cyclophosphamide 750 mg/m^2^ iv,d1; doxorubicin 50 mg/m^2^ iv,d1; vincristine 1.4 mg/m^2^ iv,d1; prednisone 100 mg/d po d1-5) combined with rituximab due to poor economic conditions and then underwent CHOP regiment alone for three cycles since 19th December, 2012. The follow-up computed tomography (CT) scan was given after treatment to assess the efficacy of complete remission (CR). The patient was given three-dimensional conformal radiotherapy targeted to the involved field since 14th March, 2013. The radiotherapy range included the bilateral neck and bilateral supraclavicular/infraclavicular area. The prescribed dose was 40 Gy in 2.0 Gy fractions daily; five fractions a week were usually used. The radiotherapy was ended on 17th April, 2013. The dose parameters of lung tissue were shown in Table 1. On May 23, 2013, the patient developed a low-grade fever, a dry cough, and dyspnea. The auscultation showed coarse breath sounds in double lungs, without rhonchus and moist rales. A chest CT scan demonstrated the presence of diffuse reticular interstitial processes and honeycombing in both lungs (Figure 1(a); Figure 1(b)). The blood and sputum cultures were both negative. The blood routine examination was normal. After consultation by respiratory department experts, the pulmonary infection was excluded, then acute radiation pneumonitis (RP) was diagnosed, and empirical antibiotics were injected. Steroid therapy comprising methylprednisolone (60 mg bid) was administered for inflammatory lung disease. Three days later, these symptoms were basically relieved. The follow-up chest CT on May 30, 2013, showed that the inflammatory lesions had been absorbed compared with the previous CT (Figure 2(a); Figure 2(b)). In view of this, the dosage of steroid was gradually reduced. The patient showed cough, aggravated asthma associated with fever again on June 7, 2013. The patient's symptoms were improved after strengthened anti-infection, methylprednisolone (80 mg bid), and supportive treatment. Then the antibiotic was withdrawn, and the dosage of methylprednisolone was gradually reduced to 10 mg bid; then she was given oral administration of prednisone tablets, and the dosage of steroid was continued to be reduced until withdrawal. The total duration of use of steroids lasted for 2 months. During the follow-up after three months, the patient was in a stable condition, without fever, cough, and dyspnea, so that she was able to be in normal life and at work.

## 2. Discussion

Radiation pneumonitis (RP) is a relatively common side effect of radiation therapy for thoracic malignancies. In severe cases, it may become life threatening. The incidence and grade of RP have been revealed to be significantly correlated with the *V*20 value (percentage of both lungs receiving >20 Gy, not including the gross tumor volume) and MLD (mean lung dose) [1, 2]. Generally, *V*20 was considered to be safe below 25~30%, and the overall treatment time is safe for small-volume lung tumors with an MLD ≤ 13.6 Gy. Yorke et al. [3] reported 10 ≥Grade 3 pneumonitis cases from 78 cases of NSCLC patients within 6 months after treatment; *V*5–*V*40 in the whole lung and ipsilateral lung were associated with the risk of severe radiation pneumonitis, and the most significant correlations were for *V*5–*V*13 in ipsilateral lung. It was considered that the occurrence of severe radiation pneumonitis was closely associated with the size of low-dose region in the ipsilateral lung. In domestic studies, Wang et al. [4] reported 161 patients with thoracic malignancies treated using three-dimensional conformal radiation therapy and concluded that *V*5 may be the most valuable predictor of RP; when *V*5 exceeds 55%, the probability of RP of grade 2 or more may increase notably. This patient was irradiated only in the double lung apex; *V*20, MLD, and *V*5 of double lungs were 7.2%, 3.6 Gy, and 14.2%, respectively. However, there was a serious radiation pneumonitis, which was then extended to the middle and lower lung. What is the main cause? The author held that the female patient with the same radiation field was more prone to reveal radiation pneumonitis due to her relatively small lung volume. This patient had the lung volume of 1074 cm^3^, much lower than the normal level. In addition, the main reason of radiation pneumonitis in this case might be the history of chemotherapy.

Radiation pneumonitis can lead to massive pulmonary consolidation and pulmonary fibrosis. The patient may die from respiratory failure. The lung is one of the most sensitive tissues to ionizing radiation. After irradiation, an immediate effect of tissue irradiation is the generation of reactive oxygen (ROS) and nitrogen (RNS) species which lead to apoptosis of types I and II pneumocytes [5]; besides, it results in the loss of epithelial or endothelial cells or vessel integrity. Pulmonary radiation also reduces microvessel density and lung perfusion and promotes hypoxia [6]. During the inflammation and repair process, radiation induces a cascade of signaling events involving numerous growth factors, chemokines, and cytokines, including interleukins, tumor necrosis factor, and transforming growth factor (TGF), and recruiting an influx of leukocytes into the lung occurs, which, along with tissue remodeling, ultimately contributes to changing the composition of the lung. Findings on a chest X-ray include diffuse haziness or fuzziness in the areas of the irradiated lung, which may coalesce to form a relatively sharp edge corresponding to the shape and size of the radiation field [7]. However, there might be a very rare situation in the clinical where the radiation pneumonitis occurred outside of the radiation field, namely, “abscopal effect,” which might be associated with the migration of activated macrophages from the radiation field to the outside.

Combination of chemotherapy and RT has been well reported increasing the risk of pulmonary injury, either sequential [8] or concurrent [9]. The targets of drugs with direct toxicity to human cells were consistent with the radioactive rays. The lung damages caused by chemotherapy drugs could be caused by direct toxicity, allergic reaction, or idiosyncrasy [10]. There were reports [11] that Taxol could not only increase the incidence of radiation pneumonitis, but also present an increasing trend of radiation pneumonitis with increasing dose (increased from 21% to 36%). This fully showed that the chemotherapy drugs themselves could cause lung damages, and the subclinical damages caused by chemotherapy drugs reduced the tolerance of radiation-induced lung damages.

The occurrence of radiation pneumonitis is a complex pathophysiological process, which is affected by many factors. At present, many risk factors for RP are identified, including combining with chronic lung disease, diabetes mellitus, low pre-RT (radiation therapy) pulmonary function, tumor located in middle or lower lobe, RT combined with chemotherapy, and absence of pre-RT lung tumor surgery, without amifostine combined with RT and some dose-volume parameters. In order to prevent or mitigate radiation pneumonia, radiation treatment planning techniques to minimize the dose to the normal lung should be used, and patient characteristics including age, sex, pulmonary function, smoking, and location of the tumor should also be taken into account. Currently, due to the widespread use of chemotherapy, radiation pneumonitis might occur during radiotherapy or after radiotherapy. Therefore, it is of importance for the early identification, diagnosis, and treatment of radiation pneumonitis. The use of antibiotic is invalid for radiation pneumonitis, while corticosteroids form the mainstay of therapy.

## 3. Conclusions

Radiation pneumonitis is very rare and easy to be neglected for patients with neck lymphoma who had undergone radiotherapy; however, we should take into account the possibility of occurrence of RP, especially when the patients had fever, a dry cough, and dyspnea.

## Figures and Tables

**Table 1:** Planning parameters of lung.

Parameters	Lung L	Lung R	Lung sum
*V*30 (%)	4.3	3.7	3.8
*V*20 (%)	6.3	8.5	7.2
*V*10 (%)	9	11.5	10.4
*V*5 (%)	12.5	15	14.2
Mean dose (Gy)	3.4	3.8	3.6
Volume (cm^3^)	452.8	621.2	1074
